# Supplementary material for: pandasPGS: a Python package for easy retrieval of Polygenic Score Catalog data
Source: PeerJ. 2025 Feb 12;13:e18985. doi: 10.7717/peerj.18985 (PMC11829626; doi:10.7717/peerj.18985)
Supplement: Supplemental Information 1 — The file contains the code and the main program output. The program output starts with "#". [file peerj-13-18985-s001.pdf]

Code used in Example 1

> Runtime environment: Python(3.11), plotnine(0.13.6), pandasPGS(0.1.0)

Step 1

```
from pandaspgs import *
from plotnine import*
```

Step 2

```
traits = get_traits(term='diabetes')
traits
# Trait is running in fat mode. It has 6 DataFrames with hierarchical dependencies.
# traits: 6 rows
# |
#  -associated_pgs_ids: 215 rows
# |
#  -child_associated_pgs_ids:294 rows
# |
#  -trait_categories: 13 rows
# |
#  -trait_mapped_terms: 69 rows
# |
#  -trait_synonyms: 66 rows
```

Step 3

```
traits.traits
#          id                  label                                  description
# 0    EFO_0000400            diabetes mellitus  A metabolic disorder characterized by abnormal
# 1    EFO_0006842  diabetes mellitus biomarker
# 2    EFO_0003770         diabetic retinopathy  A chronic, pathological complication associate
# 3    EFO_0004593         gestational diabetes  Carbohydrate intolerance first diagnosed durin
# 4  MONDO_0005147     type 1 diabetes mellitus  A chronic condition characterized by minimal c
# 5  MONDO_0005148     type 2 diabetes mellitus  A type of diabetes mellitus that is character
```

Step 4

```
score1 = get_scores(trait_id='EFO_0000400')
score2 = get_scores(trait_id='EFO_0006842')
score3 = get_scores(trait_id='EFO_0003770')
score4 = get_scores(trait_id='EFO_0004593')
score5 = get_scores(trait_id='MONDO_0005147')
score6 = get_scores(trait_id='MONDO_0005148')
diabetes_score = score1+score2+score3+score4+score5+score6
diabetes_score
# Score is running in fat mode. It has 7 DataFrames with hierarchical dependencies.
# scores:215 rows
# |
#  -samples_variants: 387 rows
#   |
#    -samples_variants_cohorts: 457 rows
# |
#  -samples_training: 123 rows
#   |
#    -samples_training_cohorts: 113 rows
# |
#  -trait_efo: 224 rows
# |
#  -ancestry_distribution: 510 rows
```

Step 5

```
pic=ggplot(diabetes_score.trait_efo)+geom_bar(aes(x='label'))+coord_flip()
pic.save(filename='Additional file 2.png',dpi=300)
```
