# Supplementary material for: pandasPGS: a Python package for easy retrieval of Polygenic Score Catalog data
Source: PeerJ. 2025 Feb 12;13:e18985. doi: 10.7717/peerj.18985 (PMC11829626; doi:10.7717/peerj.18985)
Supplement: Supplemental Information 2 — The file contains the code and the main program output. The program output starts with "#". [file peerj-13-18985-s002.pdf]

Code used in Example 2

> Runtime environment: Python(3.11), pandasPGS(0.1.0)

Step 1

```
from pandaspgs import *
```

Step 2

```
traits = get_traits(term='gestational diabetes')
traits.traits
#        id               label                            description
# 0  EFO_0004593  gestational diabetes  Carbohydrate intolerance first diagnosed durin...  http
traits.traits['id'][0]
# 'EFO_0004593'
traits.traits['description'][0]
# 'Carbohydrate intolerance first diagnosed during pregnancy. [NCIT: P378]'
```

Step 3

```
gd_pgs = get_scores(trait_id='EFO_0004593')
gd_pgs.scores
#       id        name                       ftp_scoring_file                      matches_publicati
# 0  PGS002256  GRS4_GDM  https://ftp.ebi.ac.uk/pub/databases/spot/pgs/s...         True
gd_pgs.scores['id'][0]
# PGS002256
gd_pgs.scores['name'][0]
# 'GRS4_GDM'
gd_pgs.scores['matches_publication'][0]
# True
gd_pgs.scores['trait_reported'][0]
# 'Gestational diabetes mellitus in early pregnancy'
gd_pgs.scores['variants_number'][0]
# 4
```

Step 4

```
gd_pgs.scores['publication.id'][0]
# PGP000282
gd_pgs.scores['publication.PMID'][0]
# 35073990
gd_pgs.scores['publication.date_publication'][0]
# '2022-01-24'
gd_pgs.scores['publication.journal'][0]
# 'Diabetol Metab Syndr'
gd_pgs.scores['publication.title'][0]
# 'An early prediction model for gestational diabetes mellitus based on genetic variants and cl
open_in_pubmed(gd_pgs.scores['publication.PMID'][0])
```

Step 5

```
gd_pgs.samples_variants
#    sample_number  sample_cases  sample_controls  sample_percent_male sample_age phenotyping_
# 0       671           332           339                 0.0            None          None
gd_pgs.samples_variants['sample_number'][0]
# 671
gd_pgs.samples_variants['ancestry_broad'][0]
# 'East Asian'
```

Step 6

```
gd_file = read_scoring_file('PGS002256')
gd_file
#        rsID    effect_allele other_allele  effect_weight hm_source   hm_rsID    hm_chr   hm_pc
# 0  rs10830963        G             C            1.327       ENSEMBL  rs10830963    11    927087
# 1   rs1436953        T             C            1.292       ENSEMBL   rs1436953    15    624140
# 2   rs7172432        G             A            1.283       ENSEMBL   rs7172432    15    623963
# 3  rs16955379        C             T            1.220       ENSEMBL  rs16955379    16    814893
```

Step 7

```
snp1=gd_file[['rsID','effect_allele','other_allele','effect_weight']].loc[0]
snp1
# rsID             rs10830963
# effect_allele             G
# other_allele              C
# effect_weight         1.327
# Name: 0, dtype: object
from pandaspgs.file_operation import genotype_weighted_score
genotype_weighted_score(snp1)
#   rs10830963_genotype  rs10830963_weighted_score
# 0          G/G                    2.654
# 1          G/C                    1.327
# 2          C/C                    0.000
```

Step 8

```
df_list = []
for x in range(len(gd_file)):
    snp_x = gd_file[['rsID','effect_allele','other_allele','effect_weight']].loc[x]
    df_x = genotype_weighted_score(snp_x)
    df_x['key'] = 1
    df_list.append(df_x)
from functools import reduce
combination_df = reduce(lambda x, y: x.merge(y,on='key'), df_list)
del combination_df['key']
combination_df
#    rs10830963_genotype  rs10830963_weighted_score rs1436953_genotype  rs1436953_weighted_scor
# 0          G/G                    2.654                 T/T                    2.584
# 1          G/G                    2.654                 T/T                    2.584
# 2          G/G                    2.654                 T/T                    2.584
# 3          G/G                    2.654                 T/T                    2.584
# 4          G/G                    2.654                 T/T                    2.584
# ..               ...                        ...                  ...                        .
# 76         C/C                    0.000                 C/C                    0.000
# 77         C/C                    0.000                 C/C                    0.000
# 78         C/C                    0.000                 C/C                    0.000
# 79         C/C                    0.000                 C/C                    0.000
# 80         C/C                    0.000                 C/C                    0.000
# [81 rows x 8 columns]
```

Step 9

```
combination_df['genotypes']=combination_df['rs10830963_genotype']+"-"+\
    combination_df['rs1436953_genotype']+"-"+combination_df['rs7172432_genotype']+"-"+\
                            combination_df['rs16955379_genotype']
combination_df['scores']= combination_df['rs10830963_weighted_score']+\
    combination_df['rs1436953_weighted_score']+combination_df['rs7172432_weighted_score']+\
                          combination_df['rs16955379_weighted_score']
combination_df[['genotypes','scores']].sort_values(by='scores', ascending=False)
#       genotypes     scores
# 0   G/G-T/T-G/G-C/C  10.244
# 1   G/G-T/T-G/G-C/T   9.024
# 3   G/G-T/T-G/A-C/C   8.961
# 9   G/G-T/C-G/G-C/C   8.952
# 27  G/C-T/T-G/G-C/C   8.917
# ..              ...     ...
# 53  G/C-C/C-A/A-T/T   1.327
# 71  C/C-T/C-A/A-T/T   1.292
# 77  C/C-C/C-G/A-T/T   1.283
# 79  C/C-C/C-A/A-C/T   1.220
# 80  C/C-C/C-A/A-T/T   0.000
#
# [81 rows x 2 columns]
```
