# Supplementary material for: pandasPGS: a Python package for easy retrieval of Polygenic Score Catalog data
Source: PeerJ. 2025 Feb 12;13:e18985. doi: 10.7717/peerj.18985 (PMC11829626; doi:10.7717/peerj.18985)
Supplement: Supplemental Information 3 — The file contains the code and the main program output. The program output starts with "#". [file peerj-13-18985-s003.pdf]

1. Use quincunx to convert all data in PGS Catalog to DataFrame

> Runtime environment: R(4.0.0), quincunx(0.1.7)

1.1 AncestryCategory

```
library(quincunx)
results=get_ancestry_categories()
```

1.2 Cohort

```
library(quincunx)
results=get_cohorts()
#   downloading [==========================================================>------]  91% eta:
# * Status code:     500
# * Endpoint:        https://www.pgscatalog.org/rest/cohort/all?offset=1180&limit=20&format=js
# * MIME type:       text/html
# * Response:        <!DOCTYPE html PUBLIC "-//W3C//DTD HTML 4.0 Transitional//EN" "http://www
# <html>
# <head>
# <meta http-equiv="content-type" content="text/html;charset=utf-8">
# <title>500 Server Error</title>
# </head>
# <body text="#000000" bgcolor="#ffffff">
# <h1>Error: Server Error</h1>
# <h2>The server encountered an error and could not complete your request.</h2>
# <p>Please try again in 30 seconds.
# </p>
# <h2></h2>
# </body>
# </html>
#
# * Message:         No message for error 500 yet!
#
# Error in request_all(resource_url = resource_url, base_url = base_url,  :
#   Failed to get all pages of /rest/cohort/all!
```

1.3 PerformanceMetric

```
library(quincunx)
results=get_performance_metrics()
# Error in `map()`:
# i In index: 2.
# i With name: estimate.
# Caused by error in `map.function()`:
# i In index: 1018.
# Caused by error:
# ! Can't coerce from a string to a double vector.
# Run `rlang::last_trace()` to see where the error occurred.
# Warning message:
# Automatic coercion from integer to character was deprecated in purrr 1.0.0.
# i Please use an explicit call to `as.character()` within `map_chr()` instead.
# i The deprecated feature was likely used in the tidyjson package.
#   Please report the issue at <https://github.com/colearendt/tidyjson/issues>.
# This warning is displayed once every 8 hours.
# Call `lifecycle::last_lifecycle_warnings()` to see where this warning was generated.
```

1.4 Publication

```
library(quincunx)
results=get_publications()
```

1.5 Releases

```
library(quincunx)
results=get_releases()
```

1.6 SampleSet

```
library(quincunx)
results=get_sample_sets()
# Error in `map()`:
# i In index: 2.
# i With name: estimate.
# Caused by error in `map.function()`:
# i In index: 422.
# Caused by error:
# ! Can't coerce from a string to a double vector.
# Run `rlang::last_trace()` to see where the error occurred.
```

1.7 Score

```
library(quincunx)
results=get_scores()
# Error in `map()`:
# i In index: 2.
# i With name: estimate.
# Caused by error in `map.function()`:
# i In index: 129.
# Caused by error:
# ! Can't coerce from a string to a double vector.
# Run `rlang::last_trace()` to see where the error occurred.
```

1.8 TraitCategory

```
library(quincunx)
results=get_trait_categories()
```

1.9 Trait

```
library(quincunx)
results=get_traits()
```

2. Use pandasPGS to convert all data of PGS Catalog to DataFrame

> Runtime environment: Python(3.11), pandasPGS(0.1.0)

2.1 AncestryCategory

```
from pandaspgs import *
results=get_ancestry_categories()
```

2.2 Cohort

```
from pandaspgs import *
results=get_cohorts()
```

2.3 PerformanceMetric

```
from pandaspgs import *
results=get_performances()
```

2.4 Publication

```
from pandaspgs import *
results=get_publications()
```

2.5 Releases

```
from pandaspgs import *
results=get_releases()
```

2.6 SampleSet

```
from pandaspgs import *
results=get_sample_sets()
```

2.7 Score

```
from pandaspgs import *
results=get_scores()
```

2.8 TraitCategory

```
from pandaspgs import *
results=get_trait_categories()
```

2.9 Trait

```
from pandaspgs import *
results=get_traits()
```
