# Supplementary figures and images for: pandasPGS: a Python package for easy retrieval of Polygenic Score Catalog data
Source: PeerJ. 2025 Feb 12;13:e18985. doi: 10.7717/peerj.18985 (PMC11829626; doi:10.7717/peerj.18985)

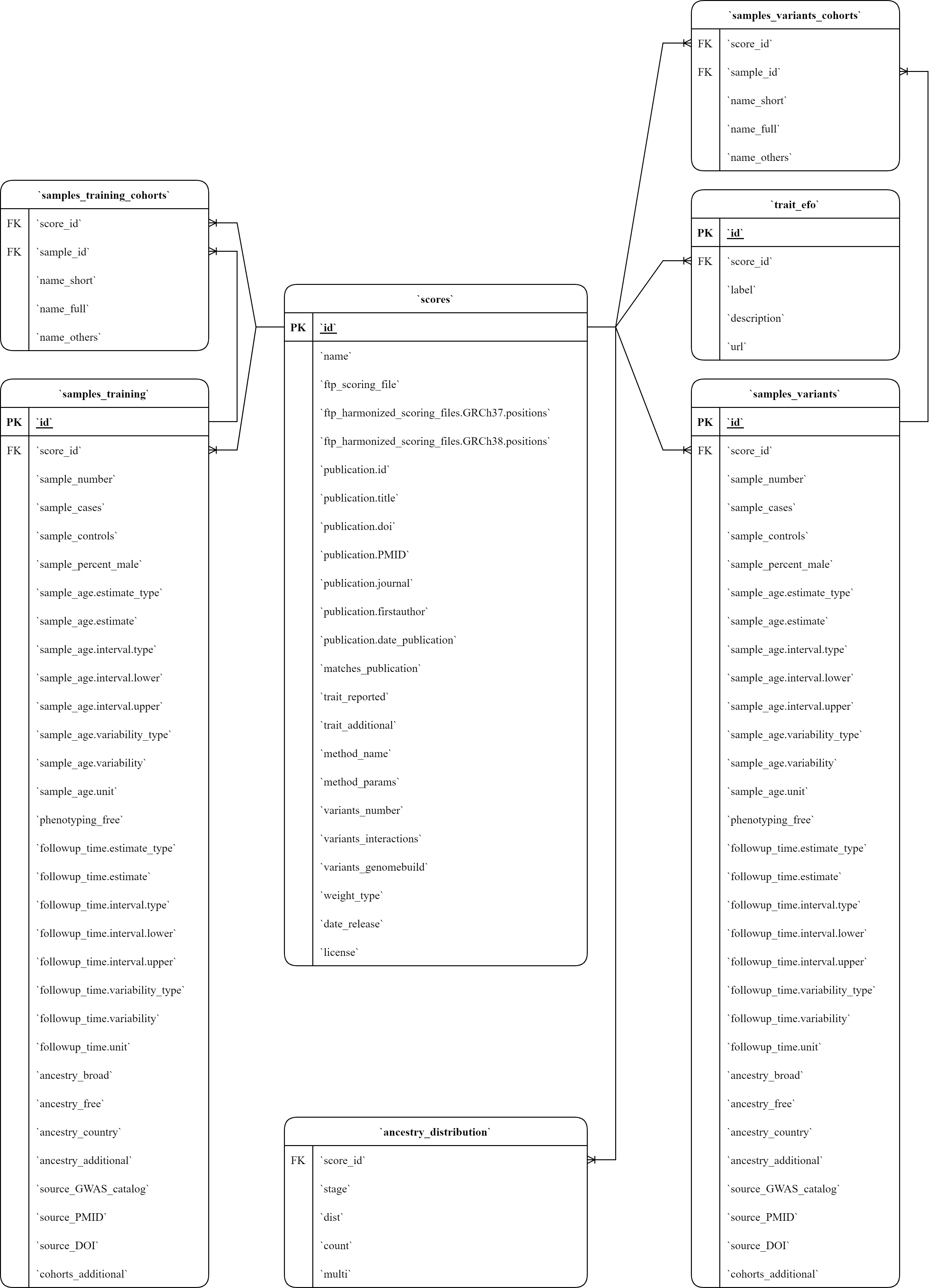

Supplement: Supplemental Information 4 [file peerj-13-18985-s004.png]

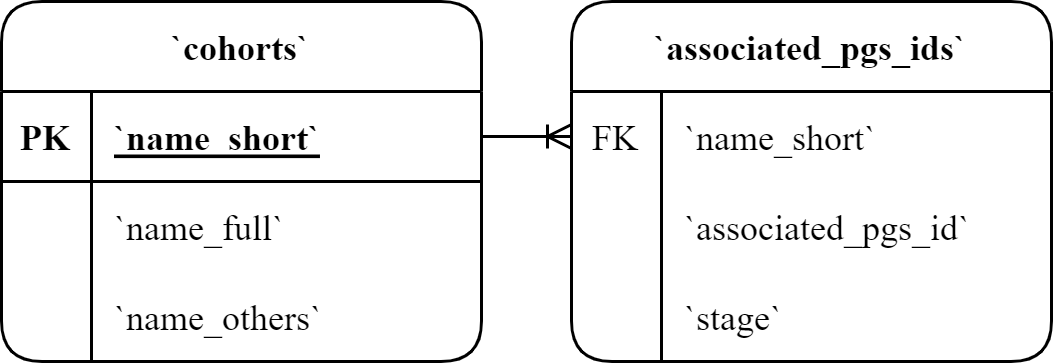

Supplement: Supplemental Information 5 [file peerj-13-18985-s005.png]

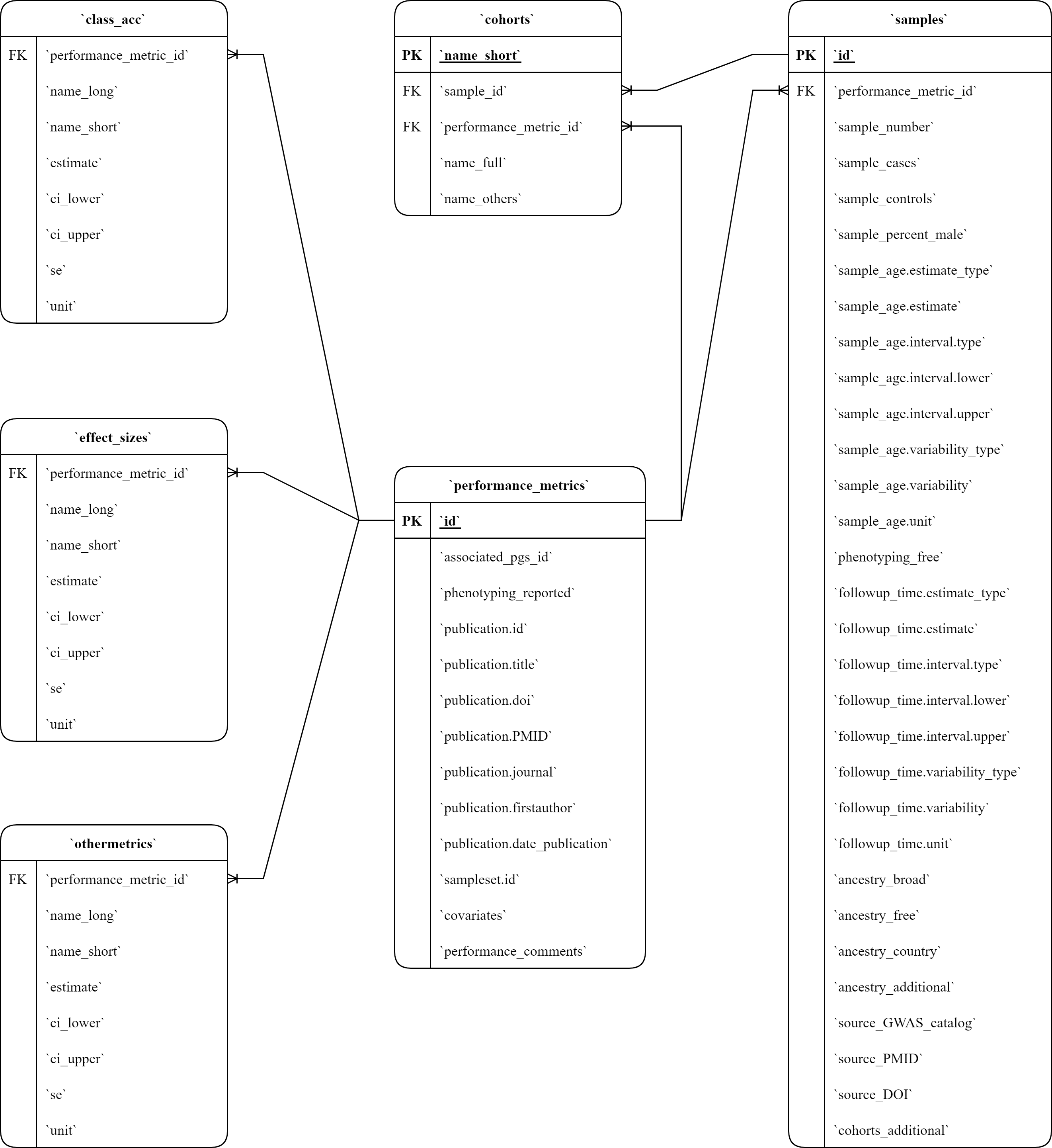

Supplement: Supplemental Information 6 [file peerj-13-18985-s006.png]

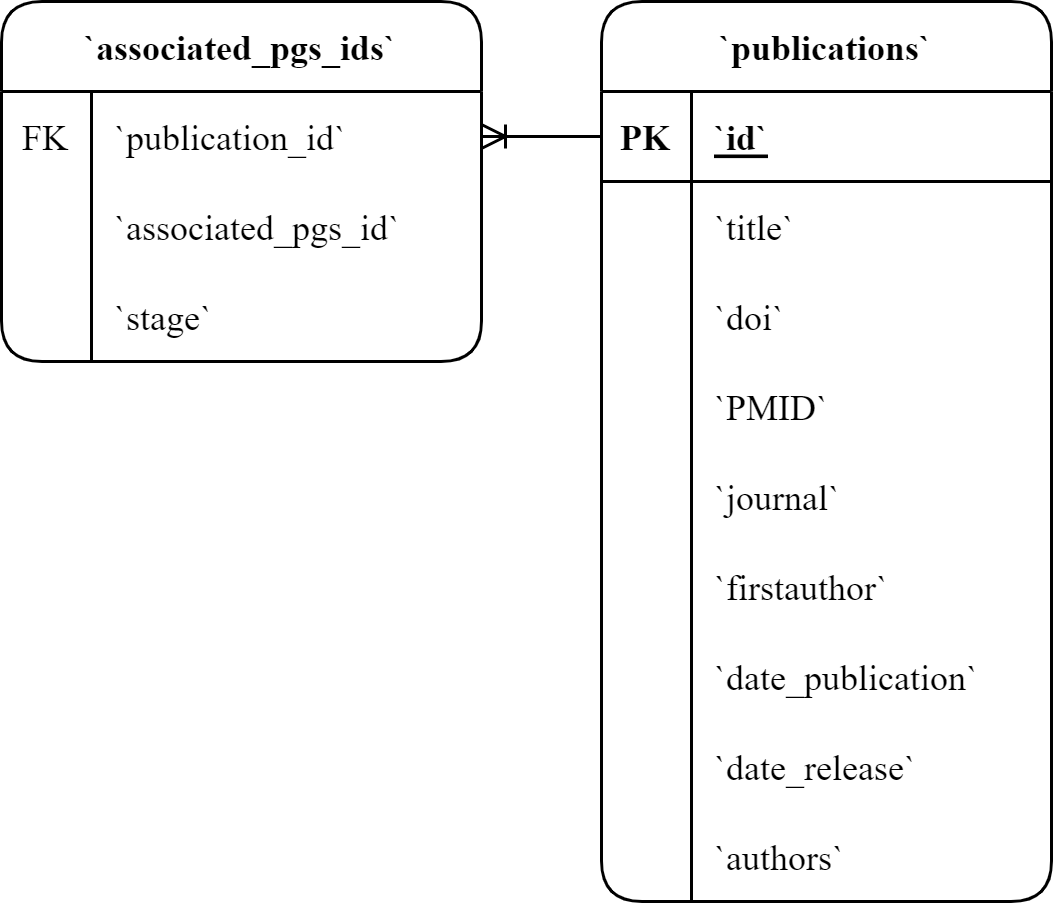

Supplement: Supplemental Information 7 [file peerj-13-18985-s007.png]

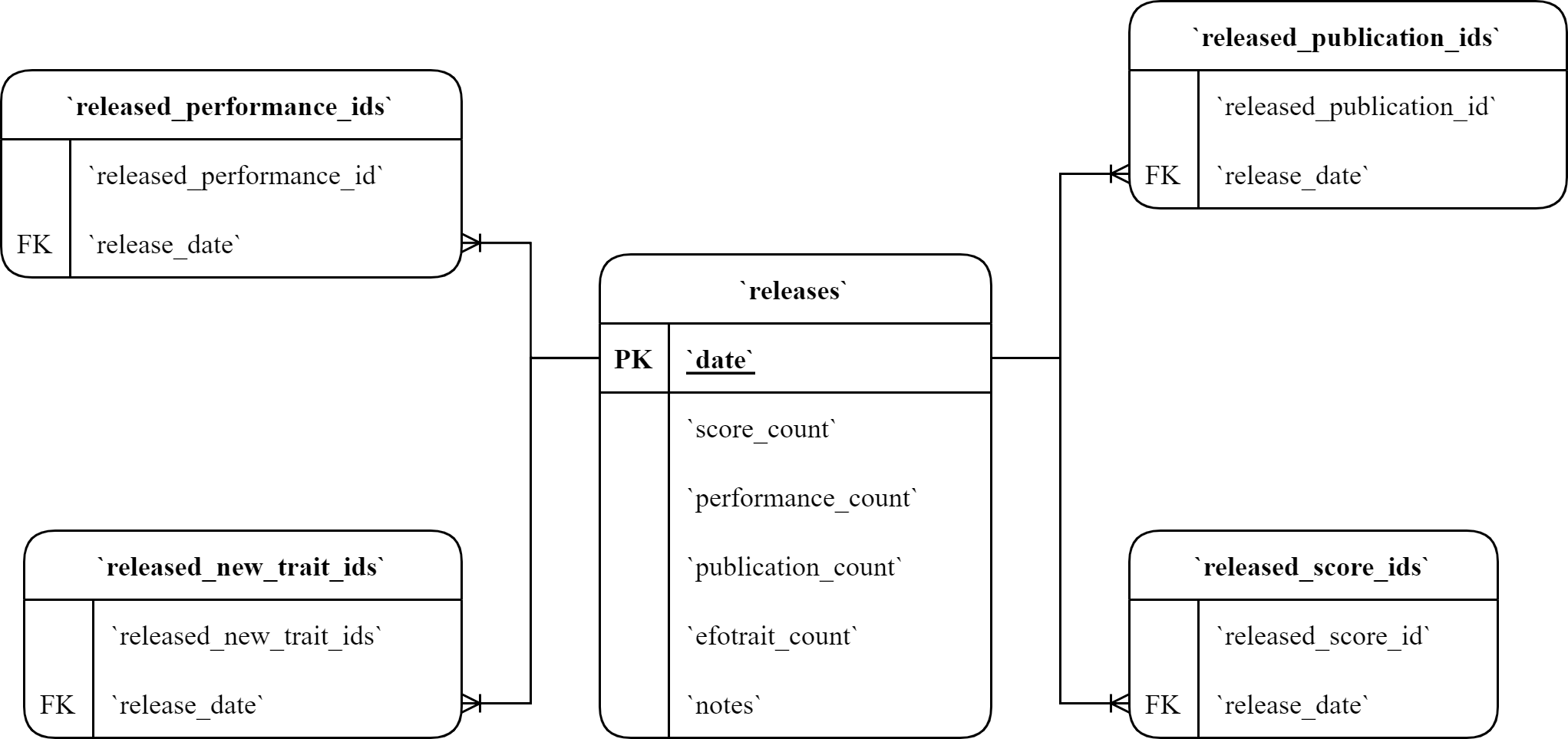

Supplement: Supplemental Information 8 [file peerj-13-18985-s008.png]

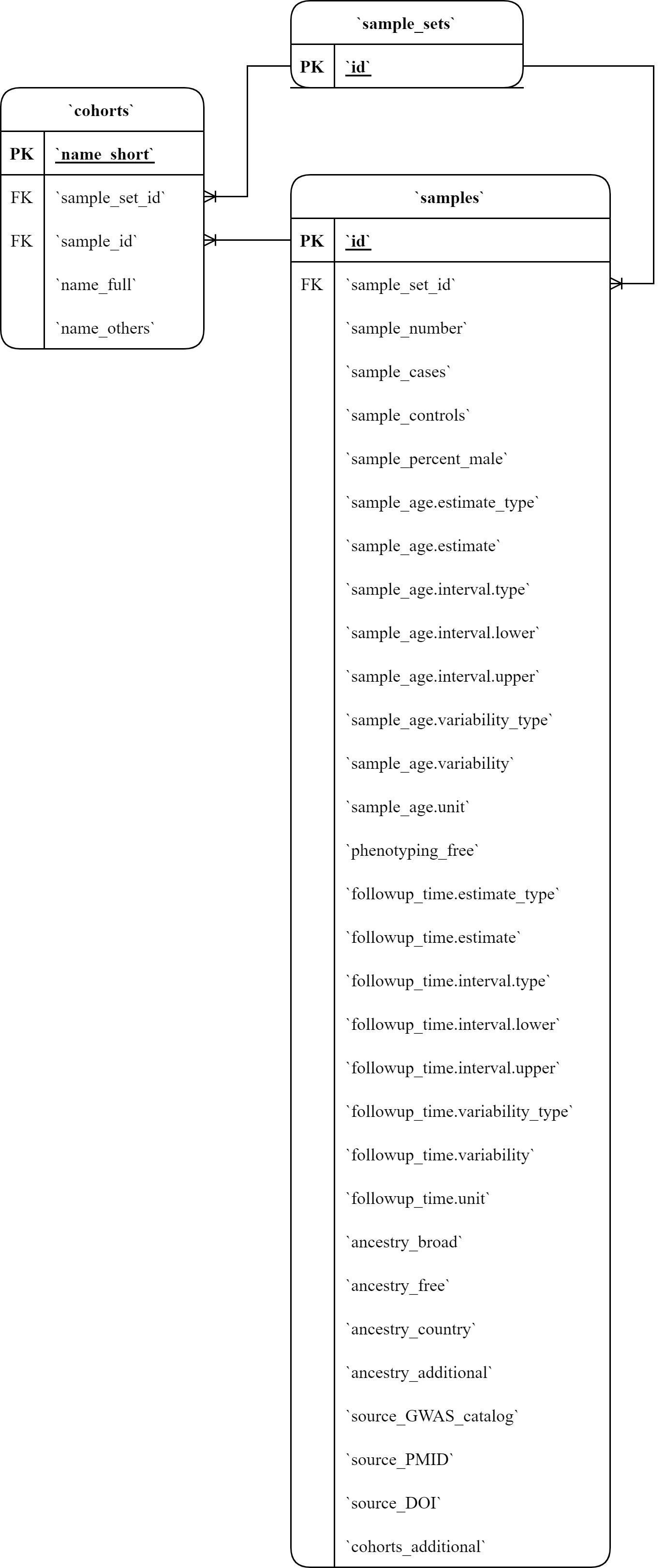

Supplement: Supplemental Information 9 [file peerj-13-18985-s009.png]

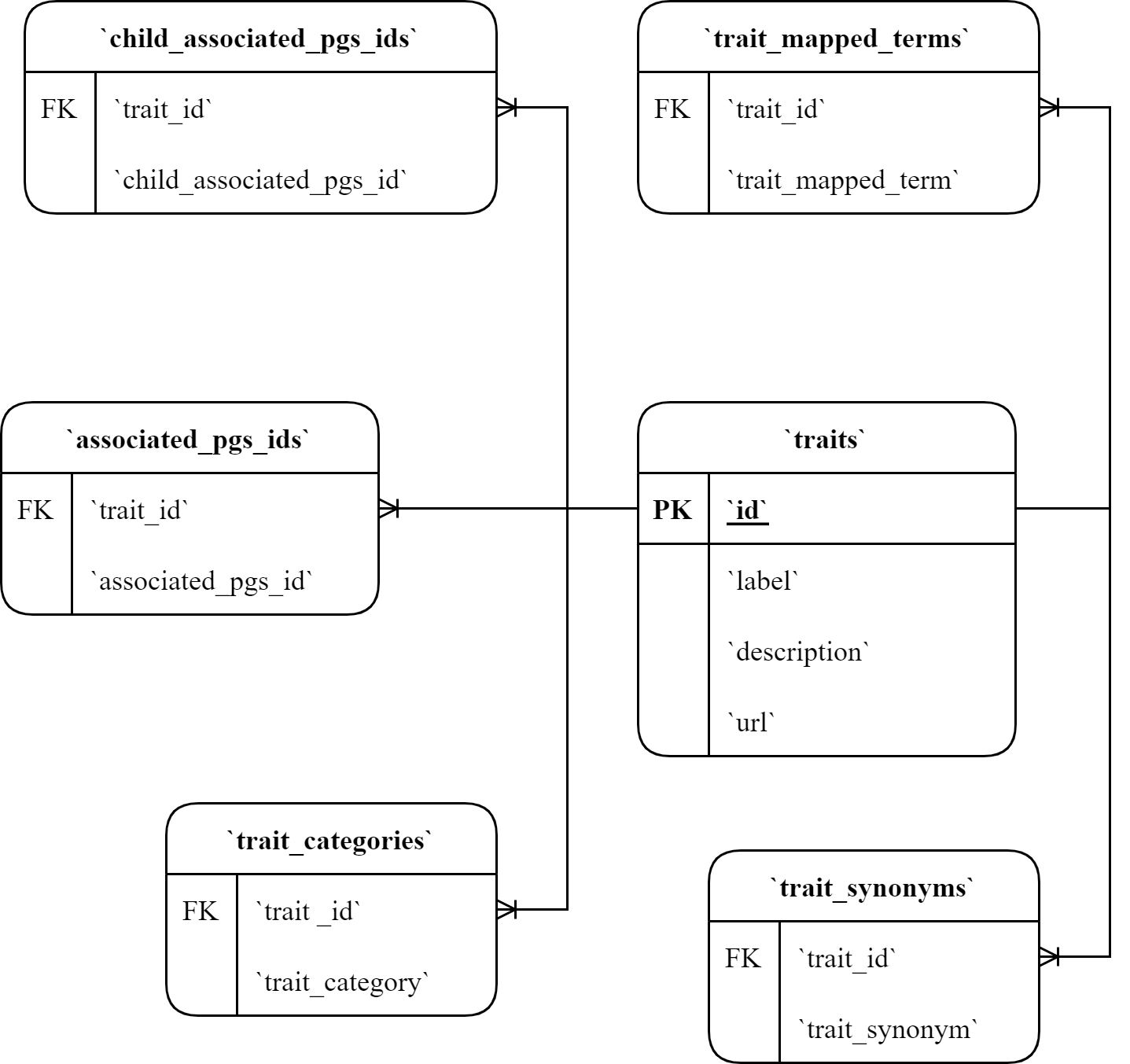

Supplement: Supplemental Information 10 [file peerj-13-18985-s010.png]

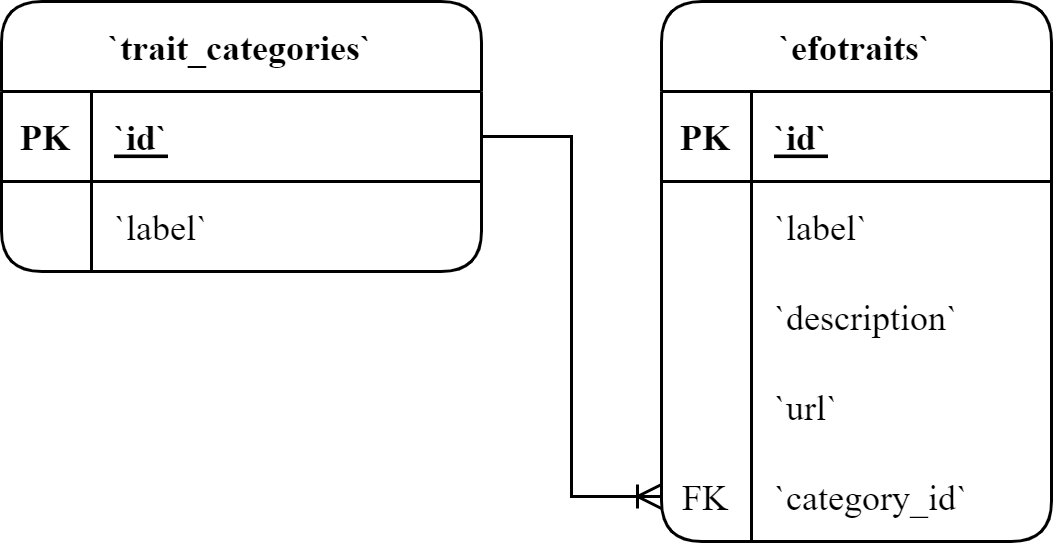

Supplement: Supplemental Information 11 [file peerj-13-18985-s011.png]

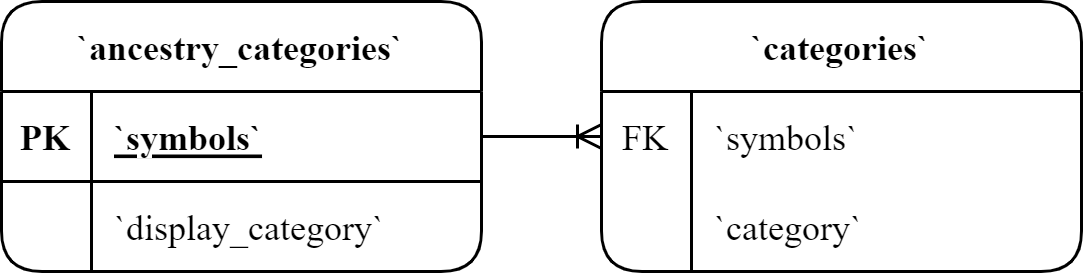

Supplement: Supplemental Information 12 [file peerj-13-18985-s012.png]
